# Initiating community engagement in an ecohealth research project in Southern Africa

**DOI:** 10.1186/s40249-016-0231-9

**Published:** 2017-03-07

**Authors:** Rosemary Musesengwa, Moses J. Chimbari, Samson Mukaratirwa

**Affiliations:** 1 College of Health Sciences, 1st Floor Desmond Clarence Building, Howard College, University of KwaZulu-Natal, Durban, 4001 South Africa; 2 School of Life Sciences, Westville Campus, University of KwaZulu-Natal, Durban, 4041 South Africa

## Abstract

**Background:**

Community Engagement (CE) in health research ensures that research is consistent with the socio-cultural, political and economic contexts where the research is conducted. The greatest challenges for researchers are the practical aspects of CE in multicentre health research. This study describes the CE in an ecohealth community-based research project focusing on two vulnerable and research naive rural communities.

**Methods:**

A qualitative, longitudinal multiple case study approach was used. Data was collected through Participatory Rural Appraisals, Focus Group Discussions, In-depth Interviews, and observations.

**Results:**

The two sites had different cultural values, research literacy levels, and political and administrative structures. The engagement process included

1. introductions to the administrative and political leaders of the area;
2. establishing a community advisory mechanism;
3. community empowerment and
4. initiating sustainable post-study activities.

In both sites the study employed community liaison officers to facilitate the community entry and obtaining letters of permission. Both sites opted to form Community Advisory Boards as their main advisory mechanism together with direct advice from community leaders. Empowerment was achieved through the education of ordinary community members at biannual meetings, employment of community research assistants and utilising citizen science. Through the research assistants and the citizen science group, the study has managed to initiate activities that the community will continue to utilise after the study ends. General strategies developed are similar in principle, but implementation and emphasis of various aspects differed in the two communities.

**Conclusions:**

We conclude that it is critical that community engagement be consistent with community values and attitudes, and considers community resources and capacity. A CE strategy fully involving the community is constrained by community research literacy levels, time and resources, but creates a conducive research environment.

**Electronic supplementary material:**

The online version of this article (doi:10.1186/s40249-016-0231-9) contains supplementary material, which is available to authorized users.

## Multilingual abstracts

Please see Additional file 1 for translations of the abstract into the five official working languages of the United Nations.

## Background

Ecohealth is a comprehensive approach to solving health problems by better understanding the connections between nature, society and health, and how drivers of social systems and ecosystems ultimately influence human health and well-being [1, 2]. The approach is based on six principles, these being systems thinking, transdisciplinarity, stakeholder participation, ecological and social sustainability, gender and social equity, and knowledge-to-action [3]. Ecohealth mostly utilises participatory action research to achieve its goals.

Participatory action research requires stakeholder involvement where all parties are expected to contribute and share control of the research process, including the formulation and implementation of actions [4]. Transdisciplinarity and stakeholder participation in the research process [4] is fraught with considerable challenges, as communities are inherently complex and dynamic. That complexity may be compounded by the need for research to be conducted at multiple sites for it to be regionally relevant and generalizable. To overcome these complexities, multicentre studies should be meticulously planned to ensure that every aspect is standardized and that protocols suit the study sites, with their socially and culturally diverse participants [5].

The planning that goes into all studies includes the process of community engagement with the selected study communities. Generally, the term community engagement (CE) is used to describe a plethora of activities that include information delivery, consultation, collaboration in decision-making, empowering action in informal groups or formal partnerships, healthcare delivery and promotion, interaction with various stakeholders, negotiation of agreements with local authorities and seeking guidance from community leaders [6–8]. The working definition developed by the Centres for Disease Control and Prevention (CDC) defines CE as, “The process of working collaboratively with and through groups of people affiliated by geographic proximity, special interest, or similar situations to address issues affecting the well-being of those people.” [9]. Community engagement in health research ensures research is consistent with the socio-cultural, political and economic contexts where the research is conducted. It also demonstrates respect for, and empowerment of, communities and improves the relevance and quality of research [10]. A review of literature [6, 7, 11, 12] shows that, in most cases, community engagement is used to describe activities such as information delivery, consultation, collaboration in decision-making, empowering action in informal groups or formal partnerships and in health care delivery and promotion.

Many research projects using the ecohealth approach have been reported, but only a few of them provide details on how communities were engaged and how the engagement was sustained throughout the project’s life span [13–17]. Engagement with communities on a specific problem is often very difficult to achieve because of competing programmes like those addressing water scarcity, hunger, poverty and HIV and AIDS [4] that are more appealing to communities. Information on effectiveness of strategies used to engage communities is scanty, as it is rarely the focus of publications. Rather, the focus is on the ecohealth interventions under study [11]. A review of some global multicentre ecohealth projects revealed that engagement occurs through forming community coalitions and partnerships, forming advisory committees, creating open community fora, and standardising principles, but not in individual site activities [13–15, 18–21]. The strategy of forming partnerships with a community’s formal, informal and governmental organizations in a community was the most reported strategy for gaining entry into communities and ensured the sustainability of projects [15, 18, 19]. Effen et al. [15] and Ranjan et al. [22] report having formed partnerships with policy makers such as departments of health, meteorological services or pollution control boards to ensure that interventions were implemented and sustained over time. Sustained community engagement can also be attained through the creation of fora such as “participative spaces” and/or “participatory workshops”, where the communities have opportunities to collaboratively plan, prioritise or evaluate ecohealth projects [13, 14, 21]. Some of the projects formed “technical committees” to advise projects on the identification of community needs and resources and to ensure commitment to sustainability [20]. In the above referenced multicentre studies, the community fora ensured that the basic principles of community engagement were uniformly applied, but the actual CE activities were influenced by each community’s own norms and values.

This paper aims to outline the process of initiating community engagement in an ecohealth study and to describe the issues emerging from its development and implementation. This publication will be a reference for developing and initiating CE strategies for ecohealth projects in the future.

## Methods

This is a qualitative, longitudinal case study approach, with each study site/country constituting a case [23]. This is an appropriate design because every project site used the same community engagement strategies, thus allowing for direct comparison and contrast between cases [23].

### The ecohealth project: MABISA

This paper describes an ongoing ecohealth study entitled ‘Malaria and Bilharzia in Southern Africa’ (MABISA). The study focuses on the social, environmental and climatic change impact on vector-borne diseases. The study is being carried out in Botswana, South Africa and Zimbabwe because they have similar social-ecological systems. The sites are in remote, arid, vulnerable and research naïve environments. During the planning phase for the project it became apparent that even though some guidance [2, 24] exists for the ecohealth projects, it is not clear on exactly what constitutes CE and how an ecohealth project would successfully develop a CE strategy. The multiple ecohealth case studies and publications from the sponsors [24] of this study imply that ecohealth projects should uphold the principle of stakeholder participation of local people, but it is not clear on how the engagement process should be done. It is against this background that the CE strategies employed for the MABISA study were documented. The study commenced in 2013 and will end in September 2016. This publication is focused only on CE activities for Zimbabwe and South Africa due to the fact that, when the documentation of these CE strategies began, the Botswana sight had not yet been activated.

### Study area (case profiles)

Gwanda District (Zimbabwe) and uMkhanyakude District (South Africa) are environmentally similar in that they are both characterized by general aridness, resulting in food insecurity and, inadvertently, increased levels of vulnerability to vector-borne diseases (VBD) like malaria and schistosomiasis [25, 26]. At both sites the link between disease transmission, burden of disease, community vulnerability and climate change is not well documented in literature. Engagement of non-governmental organizations (NGOs) and government departments with these communities is centred on provision of food, water and sanitation, bed nets and the development of irrigation schemes.

Despite the communities in these sites being vulnerable and marginalized, the two significantly differ in their social, political and administrative setups. UMkhanyakude follows traditional structures and the delegated “gatekeepers” at the site are the headmen (*Nduna*) who have jurisdiction over a village. They are accountable to the chiefs, the tribal council and their communities. The MABISA project in South Africa operated in four villages, with one chief and four headmen.

Contrary to South Africa, Zimbabwe’s governance structures are centred on the political and administrative structures. The delegated “gatekeepers” in Gwanda are councilors. They are political figures who have jurisdiction over 6 to12 villages that constitute a ward. The number of villages in a ward is not fixed. In Zimbabwe, MABISA operated in three wards presided over by three councilors, two chiefs, and 38 village heads. Councilors are accountable to the Rural District Council (RDC), District Administrator and the Member of Parliament in their area. They are by default members of every developmental committee in the wards. A ward is an administrative area within a district comprising of several villages. On average, a village consists of 100 households.

### Data collection

The primary information was collected through semi-structured key informant interviews (KII), participatory rural appraisal workshops (PRAs), unstructured interviews and direct observation of the study team and the community as they interacted during study implementation. Direct observation and unstructured interviews were used to complement the KII and PRA data. Interactions between study staff and the community were observed in order to have the right interpretation of the data collected. Informants were purposefully selected, based on their involvement in the MABISA project and their knowledge about the study area. PRAs at each site were organised by the community leaders and all villages sent their own representatives.

### Sampling procedure

Informants were purposefully sampled by selecting several potential key informants, based on their involvement in the CE activities and their knowledge about the MABISA project implementation. Key informant interviews were conducted with four headmen in South Africa, three councilors in Zimbabwe, the community liaison officers for each country, two principal investigators, two country coordinators, two project team members for each country, two CAB members for each country, four community researcher assistants for each country and one nurse at each site.

Key Informant Interviews were conducted in three phases. Phase one was 3 months after the MABISA study had entered the study communities, phase two was approximately 1 year into the study implementation and the third was approximately at the 18 month stage. This was done to have a clear picture of how the proposed CE strategies were being implemented and the types of adjustments being made as the study progressed.

All 17 key informants agreed to participate and they all provided written consent. A semi-structured interview field guide was developed and in-depth face to face interviews were conducted. Interviews lasted between 40 min and an hour. The interviews were either digitally recorded or written down with the participant’s permission.

### Data analysis

Data was analysed using a cross-case synthesis approach. Similar and contrasting topic areas and themes were assessed across cases [23]. Interviews were transcribed verbatim and divided by case. Transcriptions were coded using both predetermined codes by the general CE strategy drafted by the project team as well as newly identified codes that arose during analysis.

A table for comparing data within cases was constructed to record differences and similarities in the implementation across cases. Summary statements and identified representative quotes were recorded. Validation was done by one of the MABISA researchers to ensure that interpretation was done in a logical manner and that what the authors wrote was a true record of what transpired.

## Results

### Obtaining community approvals

The process of obtaining community approvals from the two sites was different because the political and administrative structures of the sites were different in terms of the *de facto* “gatekeeper” and was apparent when seeking community approvals. Written approvals were easier in Gwanda than in uMkhanyakude. In the latter, the gatekeepers were not familiar with the requirement of issuing a letter stating that they had granted permission. One of the headmen asked,

“*Why does the university now require actual letters? We usually get notice of medical students coming to the local clinic for periods of time and we just give verbal permission. Now what has changed? Does the university no longer trust the verbal approval of the tribal council?*”

After negotiation on the wording required by the Biomedical Ethics Committee (BREC) at the University of KwaZulu-Natal, the chief gave the headmen permission to issue letters.

In contrast, in Zimbabwe the process of obtaining an approval letter was a familiar process. The administrative system of the Rural District Council (RDC) where the Councillors report has a protocol for the process for NGOs that comes into the area with developmental projects. The following of a protocol and issuance of an approval letter is a requirement for all organizations that come into the community for any activity. This process was followed by the MABISA project getting an approval letter from the RDC, DA and councillors. These letters were then taken to the National Ethics Committee (Medical Research Council of Zimbabwe) for the issuance of ethics approval letters. Despite these differences both sites issued MABISA project community approvals, which were taken to the institutional ethics committees for the issuance of full ethics approval.

### Research literacy

A distinct similarity of the communities was that both had low research literacy. The KII revealed that both communities had very minimal experience with health research. This was validated during the PRAs, where the communities stated that they were accustomed to government programmes and NGO needs assessment surveys that mainly used the information for service provision and distribution of food or mosquito nets.

The response from one of the headmen from South Africa after being asked about the community experience with research was as follows:

“…*There has been Census, Malaria Control Programme evaluations, since 1973 they test and treat people in the villages*…” (Headman #1)

In Gwanda, the councillors were also familiar with NGO project evaluations and needs assessment surveys. However, the nurses did mention that there had been few studies, especially by university students who collect secondary data for their projects, but none had been community-based research projects.

During the PRAs the community members at both sites indicated that basic research processes such as consent, data collection techniques and results dissemination were unfamiliar to them. The MABISA team explained research concepts during the PRAs so as to sensitise the community members to the type of activities the project would be carrying out in their communities. It was also agreed with community members that the MABISA team had to embark on an awareness programme with the community leaders, the community advisory boards (CAB) and the clinic nurses to ensure that they would cascade project information to the communities. This strategy was effective in keeping the communities informed about project activities and at the same time preventing confusion, myths and misconceptions on the activities of MABISA. The project held community feedback meetings twice a year during which special sessions on research processes and the rights and roles of the community were discussed.

### Establishing community advisory mechanisms

The MABISA research team explored various mechanisms to stay informed about the community’s perception of the project and to remain socially and culturally relevant. The research team proposed three known advisory strategies to the community, namely the formation of community advisory boards (CABs), having a community liaison officer (CLO) and using local community leaders (LCL). The research team was cognisant that the establishment of these mechanisms should not create another bureaucratic structure that would be in conflict with what already existed in the communities. Each community was given a choice to choose what was best for them. Communities from both countries chose to form CABs with representation of a wide range of community stakeholders.

The uMkhanyakude site chose to remain with the community liaison officer already functional and in addition formed a CAB and used the LCL as part of the informal advisory mechanism. The CLO was familiar with the site because of previous experience of working in the malaria control programme in the same area. The main role of the CLO was to introduce MABISA to the community, make appointments with the headmen of the sites for introductory meetings, organise accommodation and advise the team of the village boundaries. The introductory meetings were successful at each village mainly due to the role played by the CLO whom they felt was “one of their own”.

The study team briefed each headman on the terms of reference of the CAB [27, 28] before the selection process. Members were selected by the communities at their local meetings, known as *Imbizo*, in the absence of the MABISA team, as this was a community activity. The CAB was formed by equal membership selected from the four villages. A 12 member CAB comprising of one headman, two community leaders, three school board members, three community care givers and three ordinary community members was established.

In Gwanda once the terms of reference for the CAB were shared with the communities, they decided to form two CABs and use the LCL strategy as well, since villages are far apart. The communities that chose to have CABs decided to use existing committees, with slight modifications as required, to fulfil the requirements of a CAB. In one ward they utilised the Ward Health Team (WHT), which has close to 30 members, and they decided to use only the Village Health Workers (VHW) to form the CAB because they represented every village. However, after training the CAB was further modified to have stakeholder representatives such as teachers, extension workers, religious leaders and the councillor. The other ward used the Health Centre Committee (HCC) to form the CAB. It already had representatives of all the villages and also had a wide stakeholder representation. Once the CAB was fully functional the need for a full time CLO fell away because the community now had direct contact with the study team through the CAB and LCL.

All CABs organise biannual community feedback workshops. They disseminate information regarding the workshops at village level well in advance of the dates of the workshops. MABISA provides the CABs with technical and financial support which is disbursed quarterly to cater for their transport and food costs during meetings. The technical support is through training, when required.

### Community empowerment

One of the objectives of the MABISA project is to develop and strengthen capacities among research groups and communities to enable them to assess and mitigate population health vulnerabilities related to malaria and schistosomiasis. At the project’s inception, the project team committed to utilising participatory methods in order to empower communities, increase involvement, legitimise the project and ensure community ownership and sustainability. Strategies that the study is using to empower the communities include both engaging and training community research assistants and utilising a citizen science approach.

### Engaging community research assistants

In order to enhance community participation and involvement, communities are responsible for selecting and recruiting community research assistants (CRAs) to work with the MABISA team. This recruitment was well received by both the Gwanda and uMkhanyakude communities and they nominated CRAs and presented them to the project. For both communities, the minimum qualifications were a matric, for uMkhanyakude, and a GCE Ordinary Level for Gwanda, and that the recruits should have enough time and flexibility for part time employment.

The CRAs are responsible for assisting the MABISA team in recruiting study participants, obtaining consent, and for data collection. The CRAs also help in developing trust between the community and the researchers, as they are familiar with the local people. Hiring of CRAs assured both individual and community empowerment. The intention of the project, as was communicated during the PRA workshops, is to have these individuals remain in the community as resource persons in dealing with malaria, schistosomiasis and climate change. The principal investigator was quoted saying

*“…we recruit what we call community research assistants who remain with skills in the society for the future…”*

Unemployment was high in both communities, although some CRAs had some prior work experience. The CRAs from uMkhanyakude comprised of high school graduates, community care givers, and college students. The CRAs from Gwanda comprised of high school graduates, two former temporary teachers and one former veterinary assistant. None had ever done health research before.

The CRAs were trained to attain the required skills for the field work. The curriculum was standardized at both study sites and it included research ethics, epidemiology of malaria and schistosomiasis, basic research methods, quality control and technical skills for data collection. The technical skills imparted included, organising teamwork and daily work activities. There was also a practical component where the CRAs did role plays of the consenting process and for approaching a household. They were also taken to the sampling points (rivers) and taught how to conduct sample collection and identify vector snails and mosquito larvae. The training was carried out over a week. At both sites educational background of individuals was the major determination of the assimilation rate of information during training. The CRAs that had some prior training in the health sector, such as the community care givers in uMkhanyakude, understood concepts quicker than those that had basic high school qualifications only. At the end of the training the University of KwaZulu-Natal gave them certificates of attendance. This provided them with evidence that they had been trained in research methods and ethics for a time when other opportunities arise in their communities.

Another shortcoming was the attrition rate at both sites. In uMkhanyakude the initial CRA team had 30 people, but after 1 year there were 11 left and two new recruits had to be hired and trained. In Gwanda there were initially 15 and after 1 year only seven remained and two new recruits were hired and trained. This attrition was mainly due to them finding permanent positions elsewhere, gaining entry into tertiary institutions and a few (three in Gwanda and four in uMkhanyakude) who felt the work was not financially rewarding. However, over time the study teams devised ways of working around the issue. In uMkhanyakude the persistent drought of 2014 and 2015 reduced the number of sampling sites and meant that the numbers of days in the field per month were reduced. In Gwanda the team utilised undergraduate research assistants who were attached to the MABISA study to collect data as well.

Another shortcoming was the ethical issues related to the CRAs’ familiarity with the community members that they interviewed. In Gwanda one of the CRAs said,

*“…with my first set of questionnaires I had to interview within the village I live in and everybody kept asking me if I know what I am doing and if I could now diagnose people but diagnosing people with malaria is not part of my job as a CRA …people also try to send me with messages to the project team and I feel burdened by that sometimes…”*

In uMkhanyakude the CRAs that were already community care givers related that:

*“…the community members sometimes mix issues because occasionally the sampled house is already a patient of yours. When you ask them questions they sometimes ask why I have to ask questions yet I know them already, so I asked the MABISA team if I could swap with another CRA if I see a patient of mine…”*

Despite this challenge the CRAs are an integral part of the project team. The study team confirmed that the CRAs contribute beyond their formal job descriptions. They are able to provide local context information that helps with project implementation. The CRAs also help with community sensitization towards the project’s activities by answering questions when they arise outside the study teams’ field days. The CRAs also serve as a community resource for malaria and schistosomiasis and are invited to talk about malaria and schistosomiasis during community meetings to enhance information dissemination. To keep the CRAs motivated the field allowances were increased periodically and they received bicycles for field work so they would not have to walk.

### Utilising a citizen science approach

One of the project activities is to develop a community-centred early warning system (CBMEWS) that the community can utilise to predict weather patterns and then be able to lessen the transmission and control of malaria and schistosomiasis. In an effort to increase the community’s research literacy and translate scientific knowledge into action, the Gwanda study team decided to use citizen scientists to collect indigenous knowledge data. Citizen science involves the use of the general public in a community to collect research data collaboratively with trained scientists [19]. It is participatory in nature and it is well suited for increasing public understanding of research [19]. For example, the project utilises community elders to collect data on indicators of weather conditions that may exacerbate malaria. They are also able to indicate the plants, animals and astronomical signs that are used traditionally to predict rainfall patterns and quantities and to relate the indicators to the occurrence of malaria. To motivate participation of these elderly volunteers, the MABISA project will award the participants “citizen certificates” to recognize their efforts. They too will then become a community resource and will assist the community with weather predictions [29].

### Initiating sustainable post study activities

At the beginning of the project the study team made their intention clear of fulfilling the ecohealth pillars of knowledge to action and sustainability. The best way to do this was to ensure that once the formal MABISA project ended, the community would be able to use the skills and knowledge learnt by the CRAs and the community members to prevent and control malaria and schistosomiasis. The project principal investigator said in an interview,

*“…Empowerment is an on-going process and at one of our meetings we mentioned that we should have capacity building going on and we train the community to continue with the activities even after the research …”*

More than any other component of the training, the communities are all eager to continue with snail identification work. This is because the gap they identified from the work done by the community health workers was that they have material on the prevention and treatment of malaria and schistosomiasis, but they do not know about the vectors that transmit and carry these diseases. In uMkhanyakude they indicated that malaria and schistosomiasis affected them directly and they wanted to learn how to identify vector snails and mosquitos to prevent them from breeding and to advise their community members to avoid infected waters. In Gwanda the feeling was the same and one of the councillors was quoted as saying

*“…we appreciate the parasitology work being done by the parasitology team. We see the work as simple to implement even after MABISA with the help of the Environmental Health Technicians, so we kindly ask if we can be assisted in coming up with activities that can be used by an ordinary villager…”*

This shows that the communities were willing to continue with study activities for their own benefit. In order to ensure that most members of the community were informed, time was set aside during the PRAs and feedback workshops to do simple snail and mosquito larvae identification.

The other ongoing activity is to form partnerships and piggyback on the local NGOs’ activities. Whilst developing the CBMEWS in Gwanda, mentioned above, the team is working with NGOs in the area to incorporate the system onto their disaster risk management platform. This will allow villagers to use the early warning system for other purposes like agriculture and waterborne disease prediction. The communities have appreciated such sustainable activities because the CBMEWS is something they would continue to use even after the project’s life span. The CBMEWS was subsequently integrated into the WHT’s disaster risk management programme [29].

## Discussion

The MABISA study demonstrated that flexibility of CE activities is the critical key to success. In similar multicentre studies across African communities, CE flexibility has also been reported [5, 30]. Similar multicentre studies also emphasised on the flexibility of CE strategies so as to respect the particular community values, traditions and culture and avoid misunderstandings and social disruptions [4, 12, 31]. Another similarity with other studies across Africa is the community entry process. Most projects start with ethical approval, administrative approval, followed by permission to enter into a community through the community leaders and then finally meeting with the community members [5, 30, 32].

The other key element that led to the success of the community entry process, particularly in uMkhanyakude, was the presence of the CLO. The introductory meetings were successful at each village mainly because the communities felt that the CLO was “one of their own”. He works closely with the Malaria control programme for the health department and deals with community activities, so he was very familiar with the people. The only pitfall of having a “local” CLO was that there was favourable bias towards the chiefs that the CLO already knew and had established relationships with, but reluctance with those he was not familiar with.

The MABISA project also showed that there is no one CE activity that proved to work better than any others. The community advisory mechanisms like the CAB, CLOs and community leaders all feed into the project and none proved less effective than the other. In a recent review Tindana et al. [33] said, “…The choice of any particular approach will be determined by the goal of the engagement…”. Even the right combination of activity cannot be predetermined. Tedrow et al. [5] described a similar multicentre study where there was a “blanket” CE strategy plan, but the implementation varied across sites according to culture and pre-existing socio-economic activities within the community before the project was initiated.

The community’s research literacy and competency also played a significant role in the engagement process. When MABISA was introduced at both communities they all expressed that they had very little experience with community-based health research. This situation presented an important co-learning environment, as the community would learn from MABISA and vice versa. Even though both sites were enthusiastic about the project and opportunities to learn new things, they were hesitant to participate at first because communities were not empowered enough to participate in health research and so they requested training. Other similar projects [19, 34–37] that have embarked on increasing research competency of marginalized communities have also reported that increasing research literacy and competency through citizen science, training in research methods and participatory activities has empowered communities greatly. Goodman et al. [34] described the teaching of research to communities as a transformative process that increases communication and develops a common language, forming equitable community academic partnerships. Research naïve communities can only learn how to work with academic researchers by being engaged in research with them. That means this “empowerment” activity is solely based on trust and mutual understanding. The researchers have to exercise integrity if they are the first research project to introduce concepts like individual informed consent, CABs, PRAs and large community feedback workshops. However, this scenario brings about a level of conflict of interest and the researchers had to emphasise that what the communities had been taught was just one way of conducting research and other projects might come with different approaches, so the communities needed to have an open but cautious mind at all times [30, 34, 38].

What was clear for MABISA was that the initial community entry and formative research activities such as the PRAs laid the foundation for all other CE activities. In order to develop a socially and culturally relevant CE strategy, it is important to carry out community diagnosis. The general MABISA CE strategy was similar in principle, but its implementation differed according to the site’s needs.

### Enablers and barriers of success

#### Flexibility

Flexibility was both enabling and a barrier, depending on the site. Each site was unique and the strategies described above did not happen in a linear or a sequential pattern at any one site. Even at site level the villages in uMkhanyakude and the wards in Gwanda responded differently in terms of time and uptake of strategies. Initially the MABISA team assumed that since the CE strategies had been jointly developed with the community, it would be easy to implement. This was not always the case. The engagement of the CRAs brought some conflict between the researchers and the community, as the project could not employ all young, unemployed people in the villages. The community automatically assumed that MABISA would keep hiring and training as the project lost some CRAs, but as the study progressed the team found new ways of coping with the loss of CRAs. In uMkhanyakude, the study team recruited 30 CRAs from all the study villages in equal numbers between them, anticipating that the groups would work in their respective villages. However, the team later negotiated that the CRAs work across the villages. As some of CRAs left and climatic conditions such as persistent drought reduced the number of sampling sites, the number of days in the field had to be reduced. At first the community members were not in agreement. Instead they wanted the team to simply hire new people, but training new CRAs became a time consuming activity so there were negotiations for combining work areas versus hiring more CRAs. In Gwanda, where the loss of CRAs meant that the undergraduate research assistants also had to assist the CRAs with data collection, this change was also met with some resistance from the community leaders who asked if their children were not “good enough”. After negotiations and explanations the leaders then agreed to this new strategy. Flexibility on the part of the communities allowed the study to be implemented smoothly.

### Traditional leader support

Observing the interaction between the MABISA team and the community members, it was apparent that the support and buy-in of traditional, political, community, and administrative leaders led to the success of the project. In both communities the leaders themselves also actively participated in the study. In Gwanda, the mosquito light traps were first done at the councillors and CAB chairperson’s houses. They expressed that community members were not accustomed to such activities and they might fear participating since actual catching of mosquitos was something they had never seen before. They lead by example and gave the project legitimacy.

However, direct community leader involvement can also be a barrier to legitimate community involvement. Community members sometimes participate only because they are afraid of being considered as uncooperative. During the feedback workshop in uMkhanyakude one of the participants said,

*“…MABISA is the chief’s project; he is the one who knows what this project is doing. I came so that I also understand it too and so that I will not be one of those people who doesn’t know the chiefs project…I am glad I attended this workshop because it is our project as well…”*

In Gwanda, it became apparent to project team members that some of the information about the community’s perceptions of MABISA was not being passed on from the leaders to the project team and, when asked about this, one of the leaders said,

*“Sometimes as leaders we can make our own judgement about some complaints and I can give you something* (Information) *that is good and cover up some things* (complaints) *that are not good, because most of the time it’s just rumours and this can disturb your work* (MABISA project)*…”*

To reconcile these disparities, MABISA became more proactive about speaking with a wide array of community members to obtain an accurate portrayal of the community’s opinions. The leaders were briefed by the project team that it did not matter how small an issue was, everything had to be discussed first before it was dismissed.

### Community participation

The issue of working with CRAs meant, in many instances, that the project had very few challenges in accessing and working within the community because of their familiarity with the area. This, however, also came with certain ethical challenges that needed very close monitoring. The fact that some of the CRAs had existing close social relations with study participants meant that the study team had to closely monitor the CRAs, and also they, in turn, were required to declare any potential conflict of interest. In uMkhanyakude, where some of the CRAs were Community Health Workers (CHW), it resulted in some participants confusing the project with the clinic health programmes. The CRAs were required to report to the team where any such confusion was evident and the study team would revisit the household to clarify issues and verify if the participant understood the project. The CAB members were also made aware of these ethical challenges and they committed themselves to increasing community sensitisation efforts. In Gwanda the CAB members also committed to “monitoring” the CRAs to ensure that they would not put pressure on participants to be recruited just because of their familiarity. A project that is working with locals as CRAs doing data collection needs to do extensive training on research integrity so as to avoid unethical practices.

### Using existing community structures

There were challenges associated with the strategy of using existing structures. The study inherited power dynamics within both communities that were in existence prior to the commencement of the study. The CABs explained that they already had existing reporting structures within their own communities and they did not wish to change their hierarchal reporting protocols. For instance, in Gwanda, the MABISA study was requested to give the councillor the identical progress reports as those they shared with the CAB chairperson, because in the absence of MABISA the chairperson reports to the councillor in the HCC which was used to constitute the CAB. This meant that all feedback sessions had to be duplicated in order to fulfil the community’s expectations of dual reporting.

### Utilising a participatory approach

The MABISA study utilises an ecohealth approach which emphasises high stakeholder and community participation. Whilst this ensures the sustainability of activities in the long run, it is a task that takes time to introduce and implement because the research teams and the community will be at different levels of understanding. As mentioned earlier, both study sites are research naive and a lot of training and sensitisation had to be done for the project to run smoothly. There were activities that had to be rescheduled and could only commence after training had been carried out and this affected project implementation timelines.

### Language and culture

The most evident barrier for the MABISA team was the issue of language and cross-cultural interaction. MABISA, being a multicentre project, had to work across different cultures and communities. In uMkhanyakude the project staff struggled to communicate with communities at the beginning. These language and culture handicaps were reduced because some project team members were able to speak the local language and dialect. The presence of these team members who knew the culture and language seemed to provide assurance that the study did not have malicious intent. The communities expressed that they felt that the conversant team members were “one of their own” because they were familiar with local culture and traditions. The local academics were not only interpreters, but were also the mediators during meetings with the traditional leaders in the initial stages.

## Conclusions

The data we collected showed that it is important for a study to plan CE activities that will enable researchers and communities to mutually benefit from research. This strategy ensured that the fundamental ecohealth principles were fulfilled. In the MABISA study, the success of CE strategy was constrained by several factors such as the low levels of research literacy of the communities, inadequate capacity of communities to learn about conducting research, time, patience and financial commitment. Although the communities differed in their cultural values, literacy levels, political and administrative structures, the general strategy developed was very similar in principle, but the implementation and weight of various aspects differed between the two communities.

## Additional files

Additional file 1: Multilingual abstracts in the five official working languages of the United Nations. (PDF 747 kb)

## Abbreviations

CAB: Community advisory board
CBMEWS: Community based malaria early warning system
CE: Community engagement
CHW: Community health worker
CLO: Community liaison officer
CRA: Community research assistant
DA: District administrator
LCL: Local community leader
MABISA: Malaria and Bilharzia in Southern Africa
NGO: Non-governmental organization
RDC: Rural district council
VBD: Vector-borne disease
