# Supplementary material for: Initiating community engagement in an ecohealth research project in Southern Africa
Source: Infect Dis Poverty. 2017 Mar 7;6:22. doi: 10.1186/s40249-016-0231-9 (PMC5340018; doi:10.1186/s40249-016-0231-9)
Supplement: Additional file 1: — Multilingual abstracts in the five official working languages of the United Nations. (PDF 747 kb) [file 40249_2016_231_MOESM1_ESM.pdf]

Translation of the abstract into the five official working languages of the United Nations

إطلاق المشاركة المجتمعية في مشروع بحث الصحة البيئية في جنوب افريقيا

روزماري موسيسينجوا و موزس جي شيمباري و سامسون موكاراتيروا

ملخص
خلفية: المشاركة المجتمعية في البحث الصحي تؤكد أن البحث متسق مع السياقات الاجتماعية الثقافية والسياسية والاقتصادية التي أجري البحث عليها. التحديات الأكبر للباحثين هي الجوانب العملية للمشاركة المجتمعية في البحث الصحي المتعدد المراكز. تصف هذه الدراسة المشاركة المجتمعية في مشروع بحث الصحة البيئية المجتمعية من خلال التركيز على مجتمعين ريفيين بسيطين وضعيفين.
وسائل: تم استخدام دراسة نوعية متعددة الحالات وطويلة المدى، تم جمع بيانات من خلال تقييمات ريفية تشاركية ومجموعات نقاشية مركزة و مقابلات معمقة ومراقبات.
نتائج: يملك الموقعين قيماً ثقافية ومستويات بحث معرفي وهياكل سياسية وإدارية مختلفة. تضمنت عملية الالتزام:
1- تعريفات بالقادة السياسيين والإداريين للمنطقة 2- - تأسيس آلية استشارية مجتمعية 3- تمكين المجتمع و 4- إطلاق نشاطات مستدامة لما بعد الدراسة. وظفت الدراسة في كلا الموقعين موظفي اتصال مجتمعي لتسهيل الدخول إلى المجتمع والحصول على الأذونات. اختار كلا الموقعين تشكيل مجالس استشارية مجتمعية كآلية استشارية أساسية لهم إلى جانب استشارة قادة المجتمع بشكل مباشر. تم تحقيق التمكين من خلال تعليم أعضاء المجتمع العاديين في لقاءات تعقد مرتين في العام وتوظيف مساعدي البحث المجتمعي واستخدام العلم المدني. تمكنت الدراسة من خلال مساعدي البحث المجتمعي ومجموعة العلم المدني بإطلاق نشاطات سيظل المجتمع يستخدمها بعد انتهاء الدراسة. تم تطوير استراتيجيات عامة متشابهة في المبدأ ولكن التطبيق والتشديد على الجوانب المتنوعة اختلف في كلا المجتمعين.
خلاصات: استنتجنا أنه من المهم أن يكون التزام المجتمع متسقاً مع قيم المجتمع وسلوكه والأخذ بعين الاعتبار بمصادر المجتمع وإمكانياته. استراتيجية المشاركة المجتمعية القائمة على الانخراط الكامل للمجتمع تقيدت بمستويات البحث المعرفي المجتمعية والوقت والمصادر ولكنها خلقت بيئة بحثية مساعدة.

Translated from English version into Arabic by sjaatoul, through

TRANSLATORS
WITHOUT BORDERS

非洲南部开展社区参与生态健康研究项目

Rosemary Musesengwa, Moses J Chimbari and Samson Mukaratirwa

摘要
引言：健康研究中的社区参与（CE）确保研究与当地社会文化、政治经济背景相协调。研究人员面临的最大挑战是多中心健康研究的 CE 实践方面。本研究描述了一个以生态健康社区为基础的研究项目的 CE，重点关注两个脆弱和未曾研究过的农村社区。
方法：本研究使用定性、纵向多案例研究方法。通过参与式农村评估、专题小组讨论、深入访谈和观察法收集数据。

结果：这两个研究地区具有不同的文化价值观、研究文化水平和政治行政结构。参与过程包括：1）介绍该地区的行政和政治领导人；2）建立社区咨询机制；3）社区授权和 4）启动可持续的后研究活动。该研究在两个社区都聘用社区联络员，以方便进入社区和获得许可证。这两个社区均以社区咨询委员会作为主要的咨询机制，同时提供社区领导人的直接建议。在半年一次的会议上，通过对普通社区成员的教育，聘用社区研究助理和利用公民科学进行授权。借助研究助理和公民科学小组，本研究成功地开展了社区参与活动，并且在研究结束后仍将继续运行。两个社区的总体战略在原则上类似，但各个环节的实施和侧重点有所不同。
结论：我们认为，社区参与需与社区居民的价值观和态度保持一致，并要考虑到社区资源和能力。充分参与社区的 CE 战略虽受到社区研究文化水平、时间和资源的限制，但它创造了有利的研究环境。

Translated from English version into Chinese by Jin Chen, edited by Pin Yang

CDC
CHINESE CENTER FOR DISEASE CONTROL AND PREVENTION
NATIONAL INSTITUTE OF PARASITIC DISEASES

Amorçage d’un engagement communautaire à travers un projet de recherches en Écosanté en Afrique du Sud

Rosemary Musesengwa, Moses J Chimbari et Samson Mukaratirwa

EXTRAIT
Le contexte : L’engagement communautaire (EC) dans le domaine de la recherche en santé assure la cohérence des recherches avec les contextes socio-culturels, politiques et économiques dans lesquels ces recherches sont menées. Certains des plus grands défis à relever pour les chercheurs sont ceux des aspects pratiques de l’EC dans la recherche en santé multicentrique. Cette étude décrit l’EC au sein d’un projet de recherche communautaire en écosanté, concentré sur deux communautés rurales vulnérables et naïves à la recherche.
Les méthodes : Une démarche qualitative et longitudinale d’étude de cas multiples a été adoptée. Des données ont été recueillies à l’aide de diagnostics ruraux participatifs, de discussions de groupes de consultation, d’entretiens approfondis et d’observations.
Les résultats : Les deux sites avaient des différences de valeurs culturelles, de niveaux de culture de recherche, et de structures politiques et administratives. Le processus d’engagement a inclus : 1) une introduction auprès des dirigeants politiques et administratifs de la région; 2) la mise en place d’un mécanisme consultatif communautaire; 3) la mise en œuvre d’initiatives d’autonomisation communautaire et 4) le déclenchement d’activités post-étude durables. Sur les deux sites, l’étude a employé des officiers de liaison communautaires pour faciliter l’entrée dans la communauté et l’obtention de lettres de permission. Les deux sites ont choisi d’établir des comités consultatifs communautaires en tant que mécanisme consultatif principal en lien direct avec des dirigeants communautaires. L’automatisation a été atteinte par le biais d’un processus d’éducation de membres communautaires ordinaires lors de réunions semestrielles, par l’embauche d’assistants de recherche communautaires et par l’utilisation des sciences citoyennes. Grâce aux assistants de recherche et au groupe de sciences citoyennes, l’étude a réussi à initier des activités que la communauté continuera

à pratiquer une fois l'étude terminée. Les stratégies générales développées étaient très proches en principe, mais les deux communautés ne les ont pas appliquées de la même manière et n'ont pas mis l'accent sur les mêmes aspects.
Nos conclusions : Nous sommes d'avis qu'il est fondamental pour l'engagement communautaire d'être cohérent avec les valeurs et attitudes communautaires, et qu'il prenne en compte les ressources et les capacités communautaires. Une stratégie d'EC qui fait participer pleinement la communauté est entravée par les niveaux de culture de recherche, le temps et les ressources, mais elle créée un environnement propice aux activités de recherche.

Translated from English version into French by mparisot, through

Вовлечение сообщества в исследовательский проект по вопросам экологического здоровья в Южной Африке

Розмари Мусесенгва (Rosemary Musesengwa), Моисей Чимбари (Moses J Chimbari), Самсон Мукаратирва (Samson Mukaratirwa)

АННОТАЦИЯ

Обоснование. Вовлечение сообщества в исследование здоровья обеспечивает соответствие проекта социально-культурному, политическому и экономическому контексту, в котором он проводится. Главную трудность для исследователей представляют практические аспекты вовлечения сообщества в многоцентровое исследование здоровья. Данная работа описывает вовлечение сообщества в исследовательский проект по вопросам экологического здоровья в двух уязвимых общинах, не участвовавших ранее в каких-либо исследованиях.
Методы. Было проведено продолжительное качественное исследование нескольких конкретных случаев. Данные были собраны посредством оценки ситуации жителями сельской местности, обсуждения в фокус-группах, глубинных интервью и наблюдений.
Результаты. Две исследовательские площадки имели различные культурные ценности, уровень исследовательской грамотности, политическое и административное устройство. Процесс вовлечения включал: 1) знакомство с местными административными и политическими лидерами, 2) формирование общинного консультативного механизма, 3) расширение прав и возможностей общин и 4) инициирование деятельности, которая бы продолжилась после завершения проекта. Чтобы облегчить вхождение в общину и получить необходимые разрешения, в обоих случаях были наняты сотрудники по связям с общиной. В качестве основного консультативного механизма обе общины решили учредить общественные консультативные советы, а также обращаться за непосредственной консультативной помощью к своим лидерам. Расширение прав и

возможностей было достигнуто посредством обучения рядовых членов общины на встречах, проходивших два раза в год, привлечения научных сотрудников и широкого круга добровольцев. При помощи научных сотрудников и группы гражданских исследователей данному проекту удалось инициировать деятельность, которую община продолжит после его завершения. И хотя общие стратегии, разработанные в ходе исследования, в основе своей похожи, их воплощение и акценты в двух общинах оказались различны.
Выводы. Мы приходим к заключению, что вовлечение сообщества непременно должно согласовываться с общинным ценностями и установками, а также учитывать ресурсы и потенциал общины. Стратегия полного вовлечения сообщества ограничивается уровнем исследовательской грамотности, временем и ресурсами, но при этом создает располагающую исследовательскую среду.

Translated from English version into Russian by Aliaksandra Baravikova, through

TRANSLATORS
WITHOUT BORDERS

Iniciar la Participación Comunitaria en un proyecto de investigación sobre la ecosalud en el sur de África

Rosemary Musesengwa, Moses J Chimbari y Samson Mukaratirwa

RESUMEN

Antecedentes: La Participación Comunitaria (PC) en la investigación de salud asegura que los estudios son consistentes con los contextos socioculturales, políticos y económicos donde se realiza la investigación. El mayor desafío para los investigadores son los aspectos prácticos de la PC en la investigación de salud que involucran múltiples centros. Este estudio describe la PC en un proyecto de investigación de ecosalud basada en la comunidad enfocándose en dos comunidades vulnerables e inexpertas en la investigación.
Métodos: Se usó un método cualitativo y longitudinal de estudio de casos múltiples. Se recopiló la información a través de Valoraciones Participativas Rurales, Discusiones de Grupos Focales, Entrevistas Detalladas, y observaciones.
Resultados: Los dos sitios tenían distintos valores culturales, niveles de competencia de investigación, y estructuras políticas y administrativas. El proceso de participación comunitaria incluyó: 1) introducciones a los líderes administrativos y políticos en el área; 2) el establecimiento de un mecanismo de asesor comunitario; 3) el empoderamiento de la comunidad y 4) iniciar actividades sostenibles de post-estudio. En ambos sitios el estudio empleó a oficiales como intermediarios comunitarios para facilitar la entrada a la comunidad y para obtener cartas de permisión. Ambos sitios optaron por formar Juntas de Asesor Comunitario como su mecanismo principal de asesoría junto con consejos directos de líderes de la comunidad. El empoderamiento se logró por medio de educar a los miembros comunes de la comunidad en las juntas bianuales, el

empleo de asistentes de investigación de la comunidad y la utilización de la ciencia ciudadana. A través de los asistentes de investigación y el grupo de ciencia ciudadana, el estudio ha podido iniciar actividades que la comunidad continuará usar después de la terminación del mismo. Las estrategias generales desarrolladas son similares en principio, pero la implementación y el énfasis de los diferentes aspectos variaron en las dos comunidades.

Conclusiones: Concluimos que es crítico que la participación comunitaria sea consistente con los valores y las posturas comunitarios, y que considere los recursos y la capacidad de la comunidad. Una estrategia de PC que involucra enteramente a la comunidad se restringe por los niveles de competencia de investigación de la comunidad, por el tiempo y por los recursos, pero crea un ambiente conducente a la investigación.

Translated from English version into Spanish by Kate Pattison, through

TRANSLATORS
WITHOUT BORDERS
